# A Portable Setup for the Voltammetric Determination of Total Mercury in Fish with Solid and Nanostructured Gold Electrodes

**DOI:** 10.3390/molecules24101910

**Published:** 2019-05-17

**Authors:** Andrea Ruo Redda, Ornella Abollino, Mery Malandrino, Stefania Squadrone, Maria Cesarina Abete, Silvia Berto, Rosanna Toniolo, Francesca Durbiano, Agnese Giacomino

**Affiliations:** 1 Department of Drug Science and Technology, University of Torino, 10125 Torino, Italy; ruoreddaandrea@tiscali.it; 2 Department of Chemistry, University of Torino, 10125 Torino, Italy; ornella.abollino@unito.it (O.A.); mery.malandrino@unito.it (M.M.); silvia.berto@unito.it (S.B.); 3 Istituto Zooprofilattico Sperimentale del Piemonte, Liguria e Valle d’Aosta (IZSPLV), 10100 Torino, Italy; stefania.squadrone@izsto.it (S.S.); mariacesarina.abete@izsto.it (M.C.A.); 4 Department of Agricultural, Food, Animal and Environmental Sciences, University of Udine, 33100 Udine, Italy; rosanna.toniolo@uniud.it; 5 National Institute of Metrological Research, Physical Chemistry and Nanotechnology Division, 10135 Torino, Italy; f.durbiano@inrim.it

**Keywords:** mercury, gold electrodes, direct mercury analyser, on-site analysis, anodic stripping voltammetry

## Abstract

A simple procedure for field fish sample pretreatment was developed. This treatment in combination with square wave anodic stripping voltammetry (SW-ASV) with solid gold electrodes (SGE) and gold nanoparticle-modified glassy carbon electrodes (AuNPs-GCE) was applied for the determination of total mercury content. A certified reference material (CRM, *Tuna Fish BCR 463)*, ten freeze-dried samples of canned tuna and two fresh fish samples were analysed both with a bench-top voltammetric analyser after microwave digestion and with a portable potentiostat after mild eating using a small commercial food warmer. The results obtained by the two SW-ASV approaches and by a Direct Mercury Analyser (DMA), the official method for mercury determination, were in very good agreement. In particular, (i) the results obtained with in field procedure are consistent with those obtained with the conventional microwave digestion; (ii) the presence of gold nanoparticles on the active electrode surface permits an improvement of the analytical performance in comparison to the SGE: the Limit of Quantification (LOQ) for mercury in fish-matrix was 0.1 μg L^−1^ (Hg cell concentration), corresponding to 0.06 mg kg^−1^ wet fish, which is a performance comparable to that of DMA. The pretreatment proposed in this study is very easy and applicable to fresh fish; in combination with a portable potentiostat, it proved to be an interesting procedure for on-site mercury determination.

## 1. Introduction

Mercury emissions have increased above natural environmental levels due to anthropogenic practices, such as coal combustion, mining activities and industrial processes, whereas natural releases are mainly due to volcanoes [1]. 

Due to its long residence time in atmosphere, Hg has the possibility to travel throughout the world, impacting remote regions, such as the open ocean [2]. When elemental mercury is introduced into aquatic ecosystems, it is oxidised to inorganic compounds and then methylating microbes participate to form organic species, such as methylmercury (MeHg) [3,4]. In this chemical form, mercury shows a low hydrophilicity and can interact with cell membranes and proteins: the affinity with the components of living organisms leads to a bio-accumulation of these Hg species in the life forms placed at the top of the food chain [3]. For this reason, fish represents a critical source of Hg and MeHg into human diet [5]. 

For the determination of this element in fish, fishery products and other biological tissues, cold-vapour atomic absorption spectrometry (CVAAS) or cold-vapour atomic fluorescence spectrometry (CVAFS) can be used. CVAAS is used to determine total Hg in a sample at concentrations levels of at least 200 µg kg^−1^ [6,7,8]; CVAFS presents a low detection limit and it allows for performing measurements with a smaller amount of sample in comparison to CVAAS, but it is not widely used because it is rather expensive. In the last several years, a new device for a direct mercury analysis has been developed, which automatically performs both sample digestion and Hg detection by AAS, with short analysis times and a low limit of quantitation (LOQ), namely 0.010 mg kg^−1^ wet weight [9]. 

Several electrochemical methods have been developed for mercury determination in different matrices, especially in aqueous samples [10]. Focusing the attention on fish-based matrices, most of the electrochemical methods rely on anodic stripping voltammetry (ASV), involving a first step of preconcentration of Hg by reduction onto the working electrode (WE) and a second one of oxidative stripping. 

In particular, the U.S. Environmental Protection Agency (US EPA) issued a method for mercury determination by ASV using a glassy carbon electrode (GCE) modified with a gold film: this modification is very simple and permits for obtaining a low limit of detection (LOD = 0.1 µg L^−1^) and to work with a renewable surface [10,11,12]. Screen-printed carbon electrodes (SPCEs) modified with gold film have been employed, achieving LODs of 0.9 µg L^−1^ [13]. Tamer et al. used a platinum electrode modified with poly(3-hexylthiophene) obtaining an LOD of 5 µg L^−1^ [14]. In addition, carbon paste electrodes (CPEs) modified with several species able to complex and preconcentrate Hg(II) have been used. In these cases, LODs were in the range 0.18–0.5 µg L^−1^ [15,16,17]. Many studies have used gold nanoparticle (AuNPs)-modified electrodes for voltammetric sensing [18,19]. Gold is an appropriate substrate for the electrochemical determination of Hg because of its high affinity toward this element that can increase preconcentration effect [20]. Moreover, AuNPs show unique properties such as high conductivity, good biocompatibility, and large specific surface area and can bind to the surface of many polymers through covalent bonding to functional groups such as CN, NH_2_, and SH [21]: these properties are very attractive for sensors and biosensors.

The recent scientific literature continues to focus the attention on the development of new sensors for the determination of mercury in different matrices, water in particular (Table 1). Interesting chemical modifications have been proposed, but they are often not applicable for routine analysis, for mercury quantification in complex matrices.

An advantage of electrochemical analysis is that inexpensive, simple and fast measurements can be performed with miniaturized and portable instrumentation. However, most real samples have to be pretreated to dissolve the matrix and/or to extract mercury. For this purpose, in most cases, the samples have to be processed with concentrated acid extractant solutions and/or digested using a microwave oven in laboratory. Therefore, the portability of the electrochemical methods cannot be widely exploited and it is often limited to the analysis of natural waters. 

For these reasons, in this study, a simple procedure for the on-site sample pretreatment (Portable Treatment, P_T_) of fish samples was developed and the results obtained were compared to those obtained following the microwave treatment (M_T_). Mercury content in a Certified Reference Material (CRM), namely *Tuna Fish* of Community Bureau of Reference, European Commission *(BCR) 463*, in ten commercial canned tunas (CTs) after freeze-drying, in a fresh tuna fish (TF) and in a fresh swordfish (SF) was determined by ASV coupled to a solid gold electrode SGE and to a home-made AuNPs-GCE. 

The same samples were (i) treated with M_T_ and analysed using a bench-top voltammetric analyser, (ii) treated with P_T_ and analysed with a portable potentiostat.

SGE was chosen for its simplicity, ease of use and transport. A procedure for the quantification of mercury with the AuNPs-GCE had previously been developed by our research group: the electrode modification is easy and good results were obtained for the quantification of Hg, in the ngL^−1^ range, both in aqueous solutions and in (solid) certified reference materials (CRMs) [34,35]. The applicability of the AuNPs-GCE was also assessed for routine mercury determination in some freeze-dried fish samples, after acid digestion in a microwave oven [36]. The main advantages of this electrode are the possibility to work with a renewable surface and the presence of NPs, which enable fast electron transfer kinetics, increase the electroactive surface area, and reduce over-potential.

The samples were also analysed by a Direct Mercury Analyser (DMA). The results were compared with those obtained by electrochemistry in order to assess the performance of the two electrodes and the applicability of the whole field approach.

## 2. Results and Discussion

### 2.1. Monitoring of the Electrode Surface

**Figure 1:**
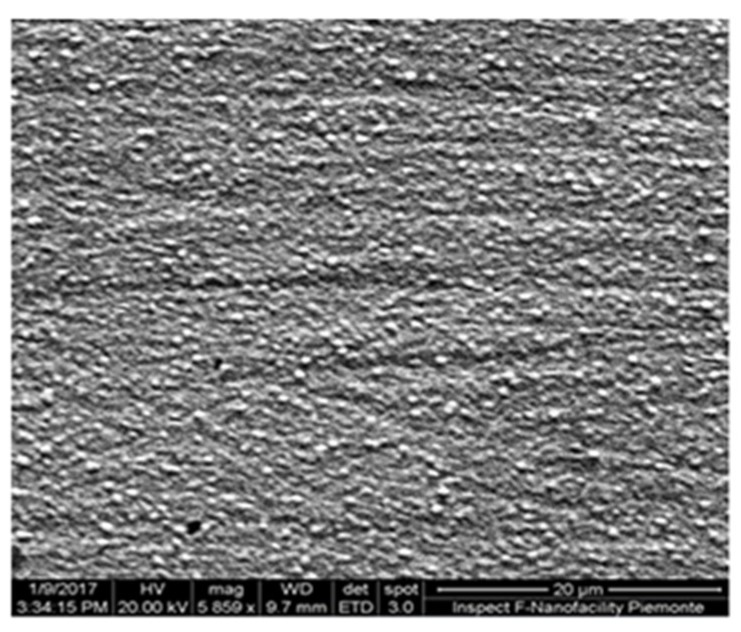
Scanning electrode microscopy (SEM) image of gold nanoparticles modified glassy carbon electrode (AuNPs-GCE) surface (This work has been performed at NanoFacilities Piemonte, INRiM, a laboratory supported by Compagnia di San Paolo).

As described in our previous papers [34,35], the deposition of AuNPs onto electrode is well visible to the naked eye through a color change of the glassy carbon surface from black to red-orange. Figure 1 shows a homogeneous gold coating composed of particles having an average diameter of approximately 100 ± 25 nm. 

A good uniformity in the distribution of the AuNPs was obtained with different GCEs and different brands of Au salts.

**Figure 2:**
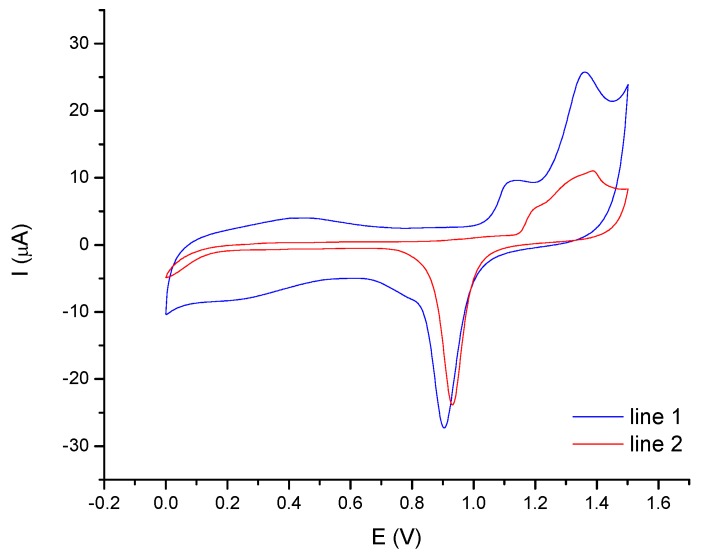
Cyclic voltammetry (CV)-voltammograms recorded in 0.5 M H_2_SO_4_ for gold nanoparticles modified glassy carbon electrode (AuNPs-GCE), line 1, and solid gold electrode (SGE), line 2.

Cyclic voltammetry (CV) was applied to daily monitor the quality of the SGE and AuNPs-GCE surface. Figure 2 shows the voltammograms recorded in 0.5 M H_2_SO_4_ solution with the two electrodes. The obtained CV profiles are well known in literature and identify the gold surface [37,38,39]. 

If the voltammogram shows additional peaks in comparison to Figure 2, a worsening of the electrode performance is observed. These peaks are caused by the formation of multilayers of oxides in which gold can exist in different oxidation states (Au0/AuI/AuIII). After the treatments with ethanol and water, a cyclic voltammogram like that reported in figure is again obtained. When no peaks appear, the electrode is not working any more. This is due to the formation of many layers of oxides on the electrode surface that form a passivating layer on the electrode surface After the mechanical polishing with alumina powder and chemical treatment with ethanol and water, the cyclic voltammogram obtained is again like that reported in the figure.

Ten CV-scans in H_2_SO_4_ were recorded before the analysis start. CV-treatment in H_2_SO_4_ is also used by many researchers as activation step for noble metals-based electrodes [40]: in our experience, it is convenient to combine this procedure with a further activation step by applying a potential of 0.60 V for 60 s in 0.06 M HCl solution before mercury determination: this step is useful for removing all naturally occurring Au oxides from the surface of the electrode. The combination of these two electrochemical pretreatments permits improving the quality and the reproducibility of Hg signal.

### 2.2. AFM Analysis

**Figure 3:**
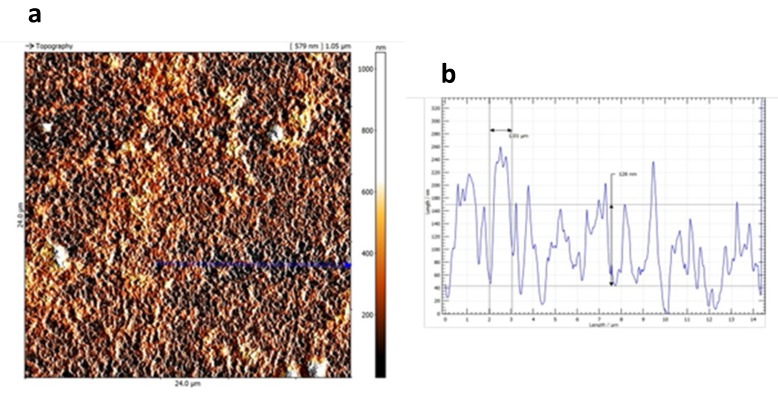
Atomic force microscopy (AFM) topographical image (**a**) and surface roughness profile (**b**) of an AuNP layer electrochemically deposited on a glassy carbon plate correspondent to the blue line present in Figure 3a.

The surface roughness of AuNPs was estimated using atomic force microscopy (AFM). Since it was not possible to directly examine the AuNPs-GCE because it does not fit to the AFM-holder, the same procedure of surface cleaning and AuNP modification for GCE was applied to a glassy carbon plate having a thickness of 3 mm. Since it is not possible to rotate the plate during the deposition as in the case of GCE, the gold solution was maintained in agitation with a magnetic stirrer. Moreover, the glassy carbon surfaces involved in the modification are quite different: GCE has a geometric surface estimated as ≈ 3.14 mm^2^ on the basis of the radius provided by the producer, while the plate has an area of 2.5 cm^2^, estimated by direct measurement. Despite differences, AFM analysis on the GC plate confirmed the presence of a homogeneous gold layer, in agreement with scanning electron microscopy (SEM) images obtained on the AuNPs-GCE. Figure 3a shows an AFM image of the plate with the AuNPs layer on the surface. Figure 3b also reports a profile of roughness referred to the “line” drawn on the image in Figure 3a: it is possible to observe the regularity of the AuNP distribution.

### 2.3. Analytical Performance of ASV Using SGE or AuNPs-GCE for Hg Determination

Previous works [34,36] report the analytical figures of merit of the procedure (repeatability, linearity, sensitivity, trueness, and detection limit) for the determination of Hg in aqueous solutions considering the two electrodes) and the investigation about possible interferences in solution. Briefly, the height of Hg peak increases by increasing deposition time: a value of 120 s was found to be the best choice for the deposition of Hg onto SGE and AuNPs-GCE, if the concentration of Hg is less than 50 µg L^−1^. Regarding SGE in a blank matrix (0.06 mol L^−1^ HCl), LOD of Hg estimated as 3*σ*_B_/slope was 0.02 µg L^−1^, linear range 0.2−100 μg L^−1^ and sensitivity, calculated as the slope of the calibration curve in this range, was 1.71 µA/(µg L^−1^). The relative error for the determination of 1 µg L^−1^ of Hg was −1%. The LOD, linear range and sensitivity (in 0.06 molL^−1^ HCl) for AuNPs-GCE were 1 ng L^−1^, 0.010−100 µg L^−1^ and 3.5 µA/(µg L^−1^), respectively. A concentration as low as 10 ng L^−1^ of Hg was quantified with a relative error of −0.8%. Considering the low analyte concentrations expected in sample solutions, the medium exchange technique was adopted to eliminate the effect of components in the sample matrix that might cause interferences in the stripping step [41]. Using this method, an improvement of Hg recovery in the CRM was observed for both electrodes.

### 2.4. Chronoamperometry 

**Figure 4:**
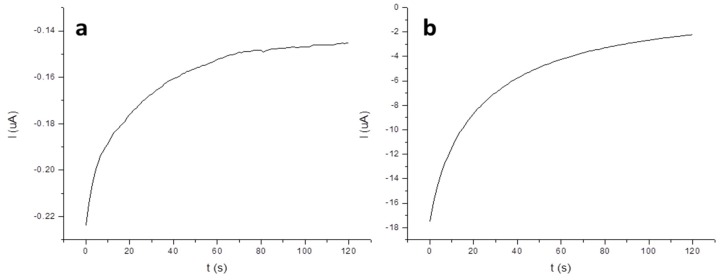
Chronoamperograms obtained for the deposition step of 5 µg L^−1^ Hg at 0 V for 120 s with (**a**) SGE and (**b**) AuNPs-GCE.

Figure 4a,b report the chronoamoperograms registered during the deposition step (0 V, 120 s) for 5 µg L^−1^ Hg at SGE and at AuNPs-GCE, respectively. The figures show that, in the case of AuNPs-GCE, the current values are lower than in the case of SGE but that the difference between the initial and the final registered current was higher due to the greater amount of the analyte deposited on the AuNPs-GCE proportional to the amount of gold surface present on this electrode.

### 2.5. Hg Determination in Fish Samples 

The concentrations found in the CRM (Certified value of Hg = 2.85 mg kg^−1^ ± 0.16 mg kg^−1^) and in the other investigated samples are reported in Table 2. In this work, the CRM and the samples were analysed also by DMA. The calibration curve obtained with DMA, y = −3.06 × 10^−7^x^2^ + 1.10 × 10^−3^x – 5.5·× 10^−3^, shows a good linearity, R^2^ = 0.9998. The results obtained by the DMA analysis were considered as a reference, taking into account its reliability, demonstrated by literature data [9] and by the excellent recovery obtained for the CRM. 

The concentration of Hg found in the samples using ASV with SGE, and with AuNPs-GCE, were expressed as percentages of recovery with respect to the results obtained by DMA. 

Regarding the sample solutions resulting from M_T_, for the CRM, recoveries of 96.8% and of 99.8% were obtained using SGE and AuNPs-GCE, respectively. In the case of the other samples, no significant mercury peaks were observed for three samples (CT3, CT4 and CT8) with the SGE, while Hg was detected in all the sample solutions with AuNPs-GCE (with recoveries between 79% and 107%). These results confirm that the larger surface area of the deposited AuNPs permits an enhancement of the sensitivity of the measurement in comparison with the one obtained in the case of a solid electrode surface. 

Regarding the analysis of the extracts obtained by P_T_, three different extraction solutions were tested on the CRM to value the best conditions to obtain a quantitative extraction in the field procedure. The obtained concentrations and the correspondent recoveries (%) were 2.66 mg kg^−1^ ± 0.22 mg kg^−1^ (93.4%), 2.58 mg kg^−1^ ± 0.43 mg kg^−1^ (90.5%) and 2.04 mg kg^−1^ ± 0.37 mg kg^−1^ (71.5%) using mixture 1: HNO_3_/H_2_O_2_ = 1:1; mixture 2: H_2_SO_4_/HNO_3_/HClO_4_ = 5:1:1 and mixture 3: HNO_3_/HCl/H_2_O_2_ = 5:1:1, respectively. 

Mixture 1 gave the best results in terms of recovery, probably because the high amount of hydrogen peroxide combined with nitric acid is very effective for the degradation of organic matter, even using a mild heating (60–70 °C). The dissolution reaction proved to be slightly exothermic and the final solution was easily filterable even with a paper filter.

Mixture 2 demonstrated a high dissolving and oxidizing capacity. The recoveries were not significantly lower than those obtained with mixture 1. Nevertheless, the dissolution reaction was strongly exothermic, and the consistency of the final solution was viscous with consequent difficulty during the filtration phase. Furthermore, it was necessary to use materials resistant to the corrosive action of the acids in use.

Mixture 3 yielded the least satisfactory results in terms of recoveries on CRM. The dissolving and oxidizing capacity was incompleted and the solution generated a lot of foam in the filtration step.

Therefore, all of the samples were digested with mixture 1 because it was the most effective in the mineralization phase and it is the easiest to use in field analysis. The samples treated with P_T_ were analysed only with AuNPs-GCE since it shows the best performance using MW_T_. In Table 2, it is possible to observe that recoveries were higher than 80% for all the samples. In particular, very good results were obtained (98% for the SF and 97% for the TF) with the fresh fish. In fact, the presence of water in the fresh samples facilitates the dissolution of the sample and the extraction of the analyte. This is a very good outcome, since the procedure was developed with the idea of applying it for on-site measurements on fresh fish.

A statistical comparison was made among the results obtained by the different techniques using ANOVA (level of probability = 95%). For all the considered samples, Hg content measured with DMA was not significantly different from those found by ASV using AuNPs-GCE. Instead, using SGE (among the quantifiable samples), the results were significantly different in one case.

The LOQ in the fish-matrix, computed as the minimum amount determined with good trueness (i.e., ≥98%), was 0.5 µg L^−1^, corresponding to 0.3 mg kg^−1^ in the fresh sample, in the case of SGE and 0.1 μg L^−1^, corresponding to 0.06 mgkg^−1^ in the fresh sample, in the case of AuNPs-GCE. 

Considering that the maximum admissible level of mercury in tuna fish is fixed to 1 mg kg^−1^_WF_ by the Commission Regulation (EU) 420/2011 [42], both voltammetric approaches can be considered suitable to monitor its content in this matrix.

**Figure 5:**
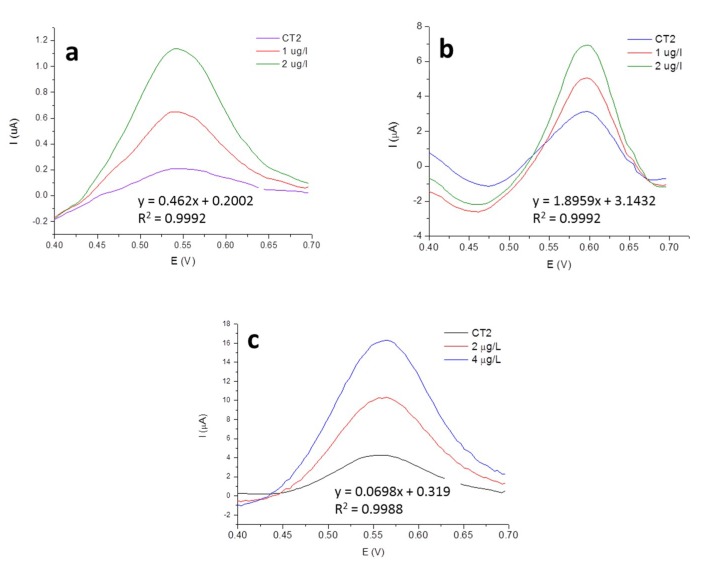
Voltammograms obtained for Hg quantification in sample CT2 using MW_T_-SGE (**a**), MW_T_-AuNPs-GCE (**b**) and P_T_-AuNPs-GCE (**c**) with the standard addition method.

Figure 5 reports the voltammograms referred to the analysis of sample CT2 using SGE voltammogram (a) AuNPs-GCE (b) or AuNPs-GCE coupled to P_T_ (c) with the two corresponding standard additions. The reported voltammograms were obtained after the subtraction of the correspondent blank signals. It is possible to observe that higher signals were recorded by AuNPs-GCE and then by SGE, even if the concentrations in the voltammetric cell are lower (the sample solutions were diluted with a different ratio in voltammetric cell, namely 1:10 in the case of SGE, and 1:20 in the case of AuNPs-GCE). 

For each determination, the corresponding equation of the calibration line and the R^2^ value are also reported: the latter show the good linearity of the technique.

### 2.6. Considerations about the Whole Portable System

- A concentration as low as 0.5 µg L^−1^ in blank solution (0.06 mol L^−1^ HCl) was quantified with three standard additions. The results obtained in the case of the benchtop and the portable instrument were 0.51 µg L^−1^ ± 0.02 µg L^−1^ (relative error: 0.03; calibration curve: y = 1.49 × 10^−5^x + 7.7 × 10^−6^, R^2^ = 0.998) and 0.48 µg L^−1^ ± 0.03 µg L^−1^ (relative error: 0.05; calibration curve: y = 2.14 × 10^−5^x + 1.02 × 10^−5^, R^2^ = 0.999), respectively.
- The autonomy of the portable battery guarantees 18 h of work; recharging the battery at night. Therefore, the possibility of working for the whole next day is guaranteed.
- Comparing the time required for MW_T_ with that for P_T_, the time determining step is the sample pretreatment, since the duration of the voltammetric analysis is the same. Digestion in a microwave oven requires about two hours for three samples in duplicate; then, the vessel have to be cleaned for the next samples’ digestion. Using P_T_, the time required for the sample pretreatment was reduced since the samples are treated in disposable Falcon tubes and it is possible to obtain three sample solutions in duplicate per hour.
- AuNPs-GCE shows better analytical performance, and in particular higher sensitivity, than SGE. However, the latter could be the best choice for on-site analysis since AuNP modification requires the use of N_2_ for the deoxygenation of the Au solution before the deposition and the obtained Au layer has to be treated with NaOH for 20 min and activated. Moreover, SGE can also be used to monitor the Hg concentration in fish, since the LOQ obtained is lower than the maximum admissible level.
- Small volumes of reagent could be adopted both for pretreatment and for the analysis step, to reduce the amount of liquid wastes. In particular, it could be very important to reduce the sample solution volume in the voltammetric cell, and consequently the volume of mercury added during the standard additions: spiked sample solutions represent a toxic waste, therefore it is fundamental to collect them and transport them to the laboratory for a proper disposal.

## 3. Material and Methods

### 3.1. Apparatus and Reagents

M_T_ of the CRM and the CT samples was carried out by a Milestone MLS−1200 Mega microwave laboratory unit (Milestone, Sorisole, Italy) in tetrafluoromethoxyl bombs.

The benchtop voltammetric analyser was a PGSTAT10 potentiostat (Eco Chemie, Utrecht, the Netherlands) coupled to a 663 VA Metrohm (Herisau, Switzerland) stand. The potentiostat was interfaced to a personal computer; and the software GPES 4.9 (Autolab Software, Metrohm, Herisau, Switzerland) was used to set up the operational conditions and to produce voltammograms. 

A small commercial food warmer (Artsana s.p.A. Como, Italy) was used for P_T_.

A PalmSens3 portable potentiostat (Thasar s.r.l, Milan, Italy) was also tested to value its applicability for on-site analysis. The potentiostat was connected to a KIA-Topolino magnetic stirrer and interfaced to a laptop computer. The software PSTrace 4.6 (PalmSens BV, Houten The Netherlands) was used to set the process parameters and record the voltammograms.

A portable battery (18 h of autonomy) was used to power the food warmer and the stirrer during the analysis.

A cell with a three-electrode configuration was adopted: a solid gold electrode (SGE) or a AuNPs-GCE prepared from a commercial Metrohm GCE (see Section 3.3) as a WE, a graphite wire as a counter electrode and an Ag/AgCl/KCl (3 mol L^−1^) as a reference electrode. 

Morphological characterisation of the electrode surfaces was performed by SEM using a Stereo scan 410 SEM Inspect F^TM^ with Field Emission Gun (LEICA Microsystems, Wetzlar, Germany). 

The surface roughness of the obtained AuNPs-GCE was investigated by AFM using a Danish micro engineering scanning probe microscope (Anton Paar, Graz, Austria), SPM (AFM/Scanning Tunneling Microscope, STM, (Anton Paar, Graz, Austria). This instrument is equipped with a DME Igloo stage with 50 µm DS95-50E SPM head for the fluid environment, integrated optical axis on cantilever and total positioning and approach control via a CCD PSU camera (DME 2350, (Anton Paar, Graz, Austria) and a fully digital hold C26 Dualscope/Rasterscope controller (Anton Paar, Graz, Austria). 

A Direct Mercury Analyser DMA-80 (FKV Srl, Torre Boldone, BG, Italy) was employed; the measurements were carried out at the Istituto Zooprofilattico Sperimentale del Piemonte, Liguria e Valle d’Aosta (IZSPLV), Torino, Italy. DMA requires regular grade oxygen as a carrier and decomposition gas. The instrument is equipped with a hollow cathode lamp (λ_Hg_ = 253.7 nm) and a Si-photodiode sensor (FKV Srl, Torre Boldone, BG, Italy).

A chemometric processing of the experimental results was performed by ANOVA (Addinsoft Inc, Long Island City, NY, USA), with an XLStat 7 software package, used as a Microsoft Excel plug-in (Addinsoft Inc, Long Island City, NY, USA). 

Ultrapure water (UPW) supplied by a Milli-Q apparatus (Millipore, Bedford, OH, USA) and analytical grade reagents (Sigma Aldrich, Milan, Italy) were used throughout.

A 1000 mg L^−1^ standard solution of mercury was prepared from HgCl_2_ in 0.012 mol L^−1^ HCl and used to obtain working standards to be added in a voltammetric cell for quantification of the analyte in sample solution.

A 65% HNO_3_/30% H_2_O_2_ = 1:1 mixture was used for the digestion of the samples in microwave oven (Milestone, Sorisole, Italy).

For P_T_, three different solutions were tested for the extraction of Hg from the fish matrix, namely, mixture 1:65% HNO_3_/30% H_2_O_2_ = 1:1 (the same as the one adopted for microwave digestion); mixture 2: 95–97%H_2_SO_4_/65% HNO_3_/70% HClO_4_ = 5:1:1 [43]; mixture 3: 65% HNO_3_/37% HCl/30% H_2_O_2_ = 5:1:1 (suggested by the producer of the microwave oven).

Calibration standards for DMA were prepared using a reference solution of 1000 mg L^−1^ Hg preserved in 5% HNO_3._ Working standards of 0.1 and 1 mg L^−1^ were prepared and preserved in 3.7% HCl and stored in amber glass vials.

In addition, 100 mg L^−1^ stock solution of HAuCl_4_^·^3H_2_O (Sigma Aldrich, >99.9% trace metals basis) in UPW was prepared for the deposition of gold nanoparticles onto the electrode. 

### 3.2. Samples and Sample Pretreatment 

The CRM *Tuna Fish BCR 463*, with a concentration of Hg = 2.85 mg kg^−1^ ± 0.16 mg kg^−1^, was employed to evaluate the efficiency and the trueness of the measurements carried out by ASV and DMA.

Ten samples of CT were purchased in local supermarkets, coded 1–10 for an univocal identification. Then, each CT was opened and the liquid drained. The samples were firstly grinded in a mortar and then freeze-dried, so as to obtain a powder, which is easier to store.

Two fresh fish slices, respectively of SF and TF, were purchased in local fish markets, and frozen in order to guarantee their maintenance over time. 

For analyses by ASV, a pretreatment of the samples was required. Aliquots of 0.5 g of the CRM and of the freeze-dried samples were transferred into the bombs and digested with a mixture of 3 mL of HNO_3_ and 3 mL of H_2_O_2_. Aliquots of 1 g were adopted for the fresh samples, due to the high amount of H_2_O present in them (about 60–70% of the total weight).

For MW_T_, the following heating program of the microwave unit was adopted: 250 W for 1 min; 0 W for 1 min; 250 W for 5 min; 400 W for 5 min; 650 W for 5 min; and ventilation for 25 min. The bombs were left to cool to room temperature before opening. 

For on-site application, the P_T_ was adopted. Aliquots of 0.5 g of each freeze-dried sample or of 1 g of fresh samples were transferred into Falcon tubes with the dissolving extraction solution (HNO_3_/H_2_O_2_ = 1:1). The mixture was allowed to react for 10 min and heated for 20 min at 60 °C by immersion in “bain-marie” in a small food warmer suitable to be powered with a simple portable battery (18 h of autonomy) for use in the field. 

In the case of MW_T_, sample solutions were filtered through Whatman 5 filters, while, in the case of P_T_, sample solutions were filtered through syringe filters to filter the aliquot of sample solution to be transferred directly into the measuring cell. Then, the solutions obtained after each pretreatment procedure were filtered and diluted to 15 mL with UPW.

In the case of DMA, the analyses were performed directly on the freeze-dried samples, without any pretreatment. 

All of the experiments were performed in duplicate and blanks were simultaneously run.

### 3.3. Deposition of Gold Nanoparticles onto the Glassy Carbon Electrode

The GCE surface was polished with Al_2_O_3_ suspension and rinsed with ethanol and water alternatively. The electrode modification with AuNPs was performed keeping the GCE at −0.8 V for 6 min in a 100 mg L^−1^ HAuCl_4_·3H_2_O solution (corresponding to 50 mg L^−1^ of Au). The nanostructured electrode was maintained in 0.1 M NaOH until use [20]. 

Before starting the analyses, ten CV scans in 0.05 mol L^−1^ H_2_SO_4_ were performed, varying the potential from 0 to 1.6 to 0 V; then, the AuNPs-GCE was activated applying a potential of 0.6 V for 60 s while it was stirred in 0.06 mol L^−1^ HCl solution (see Section 2.1). 

The presence of AuNPs onto electrode was confirmed by SEM analyses and by the response of the electrode when subjected to CV, as described in Section 2.1. 

An estimation of the roughness of the AuNPs surface was performed by AFM. 

### 3.4. Solid Gold Electrode Pretreatment

At the beginning, the SGE was polished with alumina powder and activated following the same procedure adopted for the AuNPs-GCE. During all the subsequent analyses, hereafter described, the mechanical polishing was no longer performed. 

### 3.5. SW-ASV Analysis

Aliquots of the sample solutions obtained by M_T_ or P_T_ (generally, 2 mL in the case of SGE, and 1 mL in the case of AuNPs-GCE), were transferred into the voltammetric cell and diluted to 20 mL with a 0.06 mol L^−1^ NaCl solution. 

The medium exchange technique was adopted for the ASV analyses. It consists of two steps: (1) during the electrodeposition step, the potential was kept at 0 V for 120 s; then, the “Hold” function of the potentiostat was set; (2) the sample solution was replaced with a solution of 0.06 molL^−1^ NaCl and the stripping scan in the range between 0 V and 0.75 V was performed. The same instrumental parameters were adopted using both the conventional and the portable potentiostat. The parameter values (frequency = 150 Hz, initial potential = 0 V, final potential = 0.8 V, step potential = 0.004 V, amplitude = 0.03 V, scan rate = 0.6075 V/s, stirring rate = 2000 rpm) were optimised by our research group in a previous work [22].

After each measurement, the electrode was kept at 0.80 V for 30 s in a mixture of 0.2 mol L^−1^ HClO_4_/3 mmol L^−1^ NaCl/1 mmol L^−1^ NaEDTA, in order to remove residues of mercury from its surface (Metrohm, Application Bulletin No 96/4e). 

The standard addition method was adopted for the evaluation of the concentration of mercury in all investigated samples. Well defined peaks were obtained by subtracting the blank signal from the sample solution signal. 

### 3.6. DMA Analysis

DMA was used to determine the concentration of total mercury, as reported in the U.S. EPA Method 7473 (EPA, Method 7473, 2007). Calibration graphs of 0–20 ng and 20–500 ng of mercury were created by injecting increasing volumes of 0.1 and 1 mg L^−1^ mercury standard solutions, respectively, into quartz sample boats.

DMA performs thermal decomposition, catalytic reduction, amalgamation, desorption and atomic absorption spectroscopy to treat and analyse solid or liquid samples for mercury. The process takes about 5 min. The analyses were carried out using the following parameters: drying time/temperature: 90 s at 200 °C; decomposition ramp: 120 s at 750 °C; decomposition hold: 90 s at 750 °C; catalyst temperature: 600 °C; purge time: 60 s; 12 s at 900 °C; recording time: 60 s; oxygen flow: 120 mL/min. Aliquots of each sample (about 0.1 g) were transferred into the sample boats and directly analysed without any pretreatment. 

## 4. Conclusions

Even if the ASV analysis in the field is possible thanks to the availability of the modern portable potentiostat, the pretreatment step with microwave oven does not permit the on-site extraction of the analyte. A simple treatment with a mixture 1:1 of HNO_3_:H_2_O_2_ using a commercial food warmer permits quantitatively extracting Hg from the sample and is compatible with field operation. The results obtained with the on-site procedure developed in this work are consistent with those obtained with the conventional microwave digestion coupled to a benchtop voltammetric analyser and with DMA.

The suitability of ASV both with solid and nanostructured gold electrodes for the determination of mercury in fish was demonstrated. In the case of in-cell Hg concentrations higher than 0.7 μg L^−1^, both electrodes provide very accurate determinations, while only the AuNPs-GCE permits quantifying lower concentrations, thanks to the higher surface area of the gold nanoparticles. In particular, this proposed electrode shows high sensitivity, good selectivity, long-term stability and great repeatability. Mercury detection at the AuNPs-GCE showed an LOQ in fish-matrix of 0.1 μg L^−1^ (Hg cell concentration), corresponding to 0.06 mg kg^−1^_wet fish_. The simplicity of the procedure requested for the electrode modification and the good correlation between the results obtained by DMA and ASV with AuNPs-GCE confirm that the latter can be considered an effective and an inexpensive alternative to other more common techniques for the determination of mercury in fish. Moreover, the SGE can also be used to monitor Hg concentration in fish, since the maximum admissible level of this element is higher than the LOQ value.

The proposed procedure is simple, fast and inexpensive; it can be used also by unqualified personnel. It can be applied for fast screening tests, allowing for the increase of controls on fish batches, with a view to reducing the risks to the health of consumers. The next steps will be to reduce the volume of the reagents both for the pretreatment and for the analysis step in order to reduce the liquid wastes requiring disposal.

## Figures and Tables

**Table 1:** Sample matrix, pretreatment, detection limit and linear dynamic range obtained using anodic stripping voltammetry (ASV) with different electrodes for the determination of mercury, reported in papers published in 2019.

Matrix	Pretreatment	Electrode	Method	Linear Range (µg L^−1^)	LOD (µg L^−1^)	Ref
Tap water and lake water	-	FeOOH/NPG ^a^ microelectrode	SWV	4.01–441	1.57	[22]
Fish oil	Sample was added with HCl and H_2_O_2_; sonicated—diluted	Sputtered Ag-Au-Au electrodes	DPV	0−140	0.60–140	[23]
Tap water and waste water	-	DNA-RGO ^b^@AuNR-TH-SA ^c^	DPV	0.2–40	0.04	[24]
-	-	Shrink ^d^-Induced Microelectrode Arrays ^d^	SWV	0.2−1	0.09.	[25]
-	-	[Ir(TPQ)_2_(4-EO_2_-pic)] ^e^ paper based chemosensor		-	3 × 10^−3^	[26]
Water sample	-	PXO ^f^-film modified electrode	DPV	0.13–20.5	0.04	[27]
Sea water	-	DNA/PMET-AuNPs/PGE ^g^	SWV	0.01 × 10^−11^–0.02	8 × 10^−13^	[28]
River water	-	FTO ^h^ coated with PA6/CNW:rGO ^i^	DPV	501−15044	1.4	[29]
Waste water, tap water drinking water	-	RS ^j^-gRGO ^k^-GCE	DPV	1–40	0.06	[30]
Tap water, fish oil tablet, human serum, and urine samples (spiked method)	-	Bi NPs@Gr-CNTs^i^	DPV	0.2–43500	0.04	[31]
Vegetables (cabbage and capsicum) and food products (noodles)	Samples was ashed in a muffle furnace -the ashes were dissolved in HClO_4_ and HNO_3_-diluted	SPE-p-g-C_3_N_4_/O-MWCNTs ^l^	DPV	4.8–93	0.04	[32]
Lake water and tap water and rice	Water samples were filtered—added with HNO_3_—heated to remove nitric acid. Rice samples were digested in a microwave oven with HNO_3_-diluted.	Fe_3_O_4_/F-MWCNTs-GCE ^m^	SWV	2.60–6500	0.78	[33]

^a^ nanoporous gold microelectrode; ^b^ reduced graphene oxide; ^c^ gold nanorods and thymine-Hg(II)-thymine and streptavidin; ^d^ sensor based on the heat-shrinkable polymer; ^e^ inkjet-printed phosphorescent Iridium(III) complex; ^f^ poly xylenol orange; ^g^ DNA/poly-L-methionine-gold nanoparticles/pencil graphite electrode; ^h^ fluorine tin-oxide electrode; ^i^ polyamide 6/cellulose nanowhiskers/reduced graphene oxide; ^j^ rhodamine hydrazide strip; ^k^ green reduced graphene oxide; ^i^ bismuth nanoparticles decorated graphene-carbon nanotubes nanocomposite; ^l^ screen-printed electrode-porous graphitic carbon nitride nanosheets; ^m^ magnetite nanoparticles and fluorinated multiwalled carbon nanotubes.

**Table 2:** Mercury concentrations (mg kg^−1^ of fresh weight) and recoveries (%) obtained by DMA and ASV using SGE (with MW_T_) and AuNPs-GCE (with MW_T_ and P_T_). Supporting electrolyte: 0.06 M NaCl solution.

Sample	DMA	ASV SGE MW_T_	ASV AuNPs-GCE MW_T_	ASV AuNPs-GCE P_T_
CRM (2.85± 0.16)	2.86 ± 0.06 (100%)	2.69 ± 0.04 (94.4%)	2.77 ± 0.09 (96.8%)	2.66 ± 022 (93.4%)
CT1	0.66 ± 0.02	0.56 ± 0.04 (84.0%)	0.56 ± 0.08 (84.1%)	0.58 ± 0.01 (87.1%)
CT2	0.73 ± 0.02	0.65 ± 0.001 (88.4%)	0.63 ± 0.07 (86.3%)	0.68 ± 0.02 (93.4%)
CT3	0.10 ± 0.002	<0.3 *	0.10 ± 0.01 (97.3%)	0.09 ± 0.01 (90.0%)
CT4	0.18 ± 0.01	<0.3 *	0.19 ± 0.09 (106%)	0.20 ± 0.03 (110%)
CT5	1.11 ± 0.01	1.18 ± 0.02 (106%)	0.91 ± 0.04 (82.0%)	0.89 ± 0.06 (80.1%)
CT6	0.91 ± 0.03	0.81 ± 0.01 (88.8%)	0.75 ± 0.08 (81.9%)	0.84 ± 0.01 (91.2%)
CT7	0.40 ± 0.03	0.37 ± 0.02 (91.3%)	0.41 ± 0.09 (102%)	0.36 ± 0.01 (89.7%)
CT8	0.28 ± 0.01	<0.3 *	0.28 ± 0.03 (102%)	0.26 ± 0.01 (92.1%)
CT9	1.43 ± 0.07	1.27 ± 0.06 (88.6%)	1.13 ± 0.03 (79.0%)	1.35 ± 0.02 (94.4%)
CT10	1.37 ± 0.04	1.21 ± 0.08 (88.3%)	1.31 ± 0.04 (95.5%)	1.21 ± 0.04 (88.3%)
SF	0.55 ± 0.05	0.53 ± 0.03 (97.4%)	0.54 ± 0.01 (98.7%)	0.54 ± 0.02 (98.2%)
TF	0.67 ± 0.03	0.63 ± 0.08 (93.8%)	0.65 ± 0.06 (96.4%)	0.65 ± 0.09 (96.7%)

DMA: direct mercury analyser; ASV: anodic stripping voltammetry; SGE: solid gold electrode; MWT = microwave treatment; AuNPs-GCE: gold nanoparticles modified glassy carbon electrode; PT = portable procedure; CT: canned tuna; TF: fresh tuna; SF: fresh swordfish * LOQ_SGEfresh sample_ = 0.3 mg kg^−1.^
